# Double malignancy and a mycobacterial infection in a rheumatoid arthritis patient

**DOI:** 10.15537/smj.2022.43.9.20220563

**Published:** 2022-09

**Authors:** Huda AlFaris, Sarah A. Alkathiri, Doha Babelli, Fahdah Alokaily

**Affiliations:** *From the Department of Internal Medicine (AlFaris); from the Department of Rheumatology (Alokaily), Prince Sultan Military Medical City; from the Department of Internal Medicine (Alkathiri), King Saud University; from the Department of Family Medicine (Babelli), King Fahad Medical City, Riyadh, Kingdom of Saudi Arabia.*

**Keywords:** rheumatoid, arthritis, malignancy, infection, biologic therapy

## Abstract

Rheumatoid arthritis is a common autoimmune disease. Malignancy is a serious complication of rheumatoid arthritis and its treatment. In this article, we discuss a 61-year-old woman who is a known case of Rheumatoid arthritis and secondary Sjögren’s syndrome treated with disease-modifying anti-rheumatoid drugs and multiple lines of biological therapies. She was found to have recto-sigmoid cancer, disseminated tuberculosis infection, and acute lymphoid leukemia at different intervals of treatment. Therefore, it is advisable to initiate appropriate screening programs that target high-risk people for malignancy.

Rheumatoid arthritis (RA) is a chronic, disabling illness. Controlling joints damage and synovitis, and elevating the patient’s life quality are the primary goals of RA treatment. In order to choose the adequate type of treatment, several factors must be considered. These include the severity, activity, and comorbidities of the disease.

The Institution of Biological Therapies that target key components of the immune system have reformed the treatment of RA. Despite their great value, multiple adverse effects of these agents have been described, including: malignancy, infections, bone-marrow suppression, and infusion reaction.

Although it is recommended to screen and treat infections prior to starting therapy, anti-tumor necrotic factors (anti-TNF) carry a risk for infection, especially in the first 6-12 months of initiation.^1^ Rheumatoid arthritis patients per se were 4 times more likely to have tuberculosis compared to the general population.^2^ Nevertheless, anti-TNF use is associated with even higher risk.^1^

Rheumatoid arthritis patients are at increased risk for certain types of malignancies such as lymphoproliferative disorders, lung cancer, and non-melanoma skin cancer.^3,4^ Additionally, drugs used to treat RA may contribute to the risk.^5^ Further, Sjogren’s syndrome (SS) is an additional risk factor for malignancy development. Secondary SS was also found to carry a higher risk of Non-Hodgkin lymphoma (NHL) development compared to primary Sjogren’s.

Although there is a debate on the association between anti-TNF and malignancies, there is increasing evidence that links risk of non-melanoma skin cancer with anti-TNF therapy.^4,8^

We report this case of RA to shed light on the possible complications co-existing in one patient that is treated with multiple biological agents.

## Case Report.

This is a case of a 61-year-old lady who suffers from multiple diseases such as seropositive RA, secondary SS, hypertension, and diabetes mellitus. She also has a history of pulmonary tuberculosis infection which she has been treated for twice in 1988 and 2000.

### Diagnostic assessment

She was diagnosed to have RA in 2003 according to American College of Rheumatology 1990 criteria and was maintained on methotrexate, hydroxychloroquine, and sulfasalazine, in addition to prednisolone.

### Therapeutic intervention

A few years later, her RA became active, and the decision was made to start anti-TNF. As part of routine screening, purified protein derivative was carried out and documented as positive in April 2006. When active infection was ruled out, she was started on isoniazid 300 mg daily.

One month later, etanercept was started at 25 mg twice weekly. However, it was replaced by infliximab due to failure in April of 2007.

She came to the emergency room in May of 2007 complaining of widespread abdominal pain associated with bloody diarrhea, fever, and significant weight loss. Investigations included a colonoscopy with biopsy, which came back positive for rectosigmoid adenocarcinoma stage B2 in Duke’s classification. Subsequently, she underwent rectosigmoid resection and primary anastomosis followed by chemotherapy. Also, she was started on capecitabine in August of 2007 for a total of 8 cycles.

**Figure 1:**
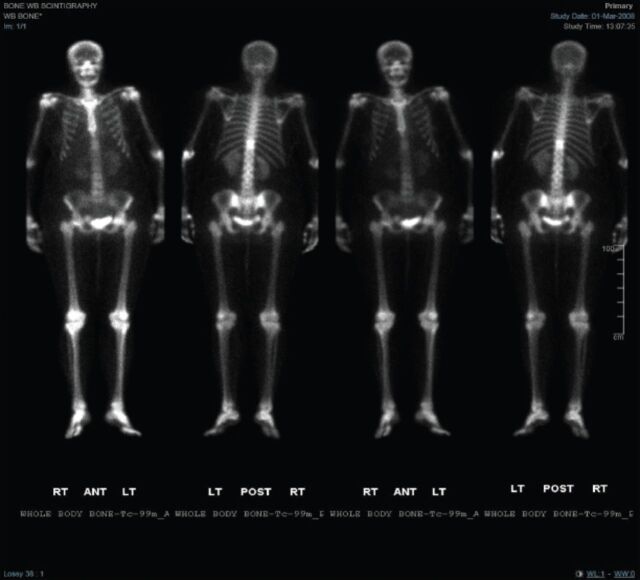
- One scanner of bone. Bone scan showed focal intense uptake noted in the region of D11.

**Figure 2:**
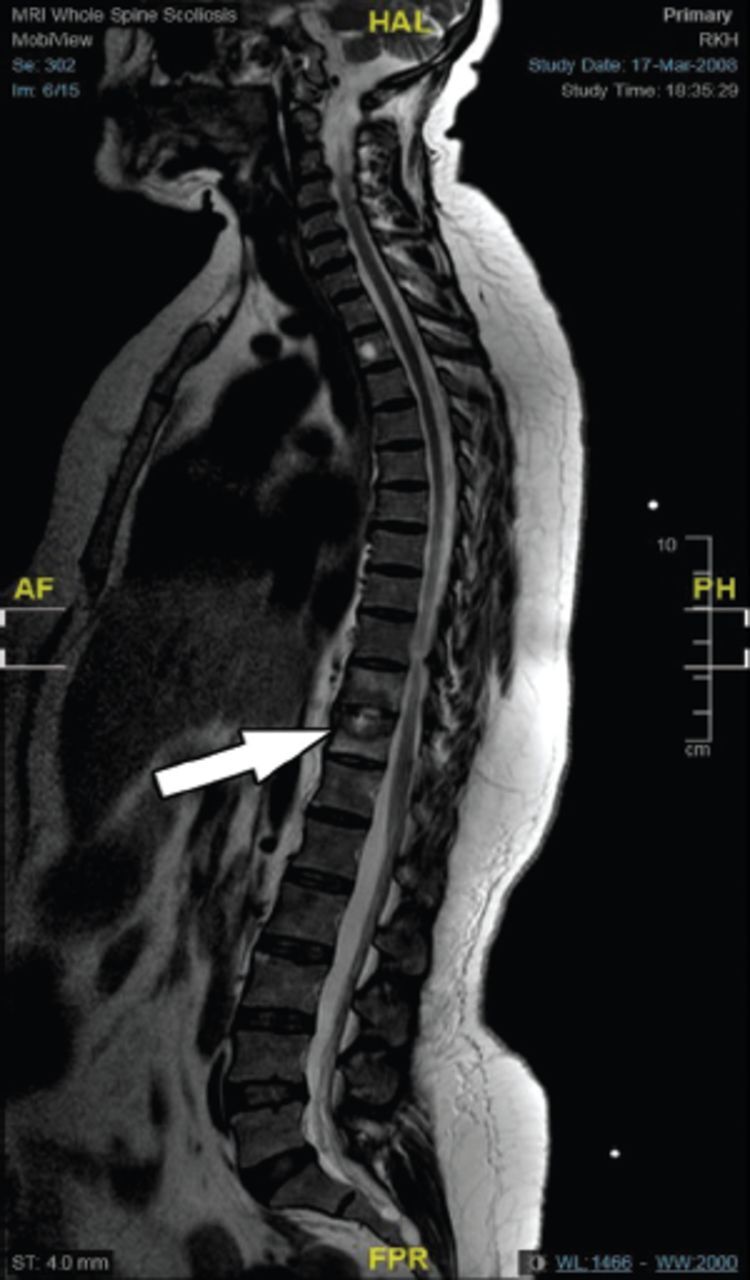
- Magnetic resonance image of the spine. D10 and D11 vertebral bodies show low signal intensity on T1 with high sginal intensity on T2 weighted image with heterogenous enhacnces on post contrast evidence of metastatic disease.

In September of the same year, she reported lower back pain and lower limbs numbness. Her computed temography scan showed an osteolytic lesion involving vertebral body D11 suspicious for metastasis. Also, a bone scan showed focal intense uptake noted in the region of D11 (Figure 2). An MRI spine report was supportive of the mentioned findings (Figure 1). In due course, she received 2 sessions of palliative radiation therapy.

### Follow-up and outcomes

A positron emission tomography scan was repeated in April of 2008 for follow-up and showed multiple suspicious lymph nodes involving the cervical area, axillary, mediastinal, inguinal, and retroperitoneal lymph nodes along with the bone disease. Ultrasound-guided fine needle aspiration of the left submandibular lymph node was carried out. The histology was consistent with granulomatous disease and AFB culture came back positive. She was therefore started on anti-TB therapy in August of 2008. The assumption of cerebral TB was made as the MRI showed brain edema as well as multiple white matter lesions. Despite this, no biopsy was obtained.

A few weeks later, she was admitted to the emergency room with signs of worsening in her general condition as well as severe lower back pain. Thus, the MRI spine was repeated on the 31st of August, 2008, and showed destructive lesions involving T10 and T11 vertebrae, along with paraspinal collection (Figure 2). The image was suspicious of an infectious process. Therefore, she underwent drainage and decompression surgery on the 25th of September, 2008. The collected samples were sent for analysis and the culture came back confirming tuberculosis.

During this period, she was maintained on methotrexate and a small dose of prednisolone, which were ineffective in controlling her RA. Therefore, a plan to start rituximab was carried out. A few months into rituximab treatment, she reported significant improvement and resolution of joint pain and morning stiffness.

She was admitted electively from the clinic for intravenous antibiotics in February of 2011 after she was found to have paraspinal area cellulitis. In July of 2012, her RA became active. Thus, rituximab was stopped and she was started on tocilizumab instead.

During the period from 2012-2016, regular follow-ups with rheumatology and oncology took place and her condition remained uneventful. However, In 2016, she was admitted to the hospital twice, the first was for abdominal wall abscess drainage and antibiotics while the second was for conservative treatment of small bowel partial obstruction.

In July of 2017, blasts were detected on her complete blood count when she was evaluated routinely before her tocilizumab dose. A bone marrow biopsy was subsequently obtained and results were consistent with precursor B cell, acute lymphocytic leukemia (ALL), and positive Philadelphia chromosome. Consequently, she was started on hyper-CVAD chemotherapy protocol in addition to dasatinib. In April 2018, she came to the clinic complaining of joint pain all over. Examination showed active synovitis, and a plan was carried out to start her on rituximab. Since then her condition remained stable.

## Discussion

We have reported a case of recto-sigmoid cancer, leukemia, and pott’s disease associated with seropositive RA. It cannot be proven, with confidence that the observed malignancies and infection were due to biologic therapies. However, developing 2 distinct types of malignancies is rare in the absence of risk factors. Therefore, another alternative explanation could be the disease nature of RA.

Askling et al^5^ have reported 20-50% risk increase for smoke related cancers and 70% increase in non-melanoma skin cancer for RA patients in comparison to the general Swedish population. On the other hand, they found 20% risk reduction of breast cancer and 25% reduction of colorectal cancer. These reduced risks are consistent with data suggesting an inverse relationship between non-steroidal anti-inflammatory drugs use colorectal cancer.^7^ Notwithstanding, the reported reduction in the risk for colorectal cancer is not in alignment with the case in discussion.

Inflammation is probably one of the most widely acceptable explanations for the association between RA and malignancy.^3^ The strong correlation between disease activity and the future risk of malignancy development might explain the protective role of corticosteroids against development of malignancy.

Smoking could also link malignancy with RA as it is considered a risk factor for both conditions. It must be stated. However, she had never smoked.

Boussios et al^6^ reported a median gap of 18.4 years (range 1-30 years) from the diagnosis of RA until the discovery of malignancy for RA. Moreover, a median time of 25.3 years (range 20-30 years) for SS was also suggested. Similarly, the odds ratio of lymphoma in SS was estimated between 2-18.8. The reported gap is rather long when compared to the subject case as the patient was firstly diagnosed with RA in 2003 and found to have colorectal cancer only 4 years later.

Several studies have examined the association between biologic therapies and malignancies. Some found no association like Askling et al.^5^ Meanwhile, Amari et al^4^ have found a higher risk of developing non-melanoma skin cancer after using anti-TNF, especially if combined with methotrexate. Longer duration of anti-TNF usage in this study was not detected as a risk factor for the development of malignancy. However, a recent observational study confirmed lack of association between the use of anti-TNF, anti-CD20, or anti-IL6 and malignancy compared to DMARDs naïve patients.^8^

Although our patient received tocilizumab later during her disease course and for a short period before she was found to have leukemia, it is worthwhile to look for the adverse effects of this agent. A systematic literature review and meta-analysis of randomized controlled trials was carried out to measure the incidence of adverse effects after treatment with tocilizumab.^9^ It was concluded that tocilizumab and methotrexate combination for RA treatment is associated with risk of infection and failed to find any rise in the incidence of malignancy, tuberculosis reactivation, or hepatitis.

Thus, several recommendations are ought to be proposed. These include carrying on age and gender appropriate cancer screening, smoking cessation, and screening for latent TB and hepatitis before the initiation of biological agents therapy. Furthermore, the compliance with prescribed treatment is an essential factor in avoiding the potentially adverse consequences of biological agents.

Rituximab may cause significant but reversible depletion on which is associated with inadequate clinical response. This explains the maintenance of remission in the last 4 years after the initial failure in 2011.^10^
